# A Portable Band-shaped Bioimpedance System to Monitor the Body Fat and Fasting Glucose Level

**DOI:** 10.2478/joeb-2022-0009

**Published:** 2022-11-04

**Authors:** Luong Duong Trong, Linh Nguyen Quang, Duc Hoang Anh, Diep Dang Tuan, Hieu Nguyen Chi, Duc Nguyen Minh

**Affiliations:** 1 School of Electrical and Electronics Engineering, Hanoi University of Science and Technology, Hanoi, Vietnam; 2 School of Biomedical Engineering, University of Sydney, NSW, Australia

**Keywords:** Bioimpedance, Home-based device, Obesity

## Abstract

With better quality of life, obesity is becoming a worldwide disease due to over-eating and sedentary lifestyle. Therefore, daily monitoring of the glucose and body fat percentage (%) is vital to keep track of one’s health. Currently, separated devices are required to monitor each parameter at home and some are still invasive to measure the glucose level. In this study, a portable band-shaped bioimpedance system is proposed to measure both parameters. The system is battery run with two main modules: the current source and the voltage recording, with minimal design to fit into a band of 150 mm x 40 mm in dimension. The impedance is measured at the frequency of 1 kHz at 30 kHz sampling frequency and in 1000 signal cycles to flatten noises. The final average impedance is calculated and evaluated in correlation with the body fat and the fasting glucose. The system was tested on 21 volunteers and 4 locations were picked for the impedance measurement: the arm under the triceps, the side of the belly, the back on one side and the thigh under the bicep femoris. The results show promising results with the arm being the best location for predicting the body fat (correlation coefficient: 0.89, 95% CI: 0.73-0.95), while the thigh impedance best correlated with the fasting glucose (correlation coefficient: 0.92, 95% CI: 0.81-0.97). These preliminary results indicate the feasibility and capacity of the proposed system as a home-based, portable and convenient system in monitoring the body fat and glucose. The system’s performance will be verified and replicated in a future larger study.

## Introduction

With the constant improving quality of life throughout the world, risks of obesity have become more and more relevant with 2.1 billion people worldwide already classified as obese or overweight [1]. The main cause of the obesity pandemic is the excessive intake of calories through daily eating while people keep burning less calories with decreasing physical activities over the past decades, i.e., less walking, cycling, sporting, etc. [2]. Besides that, medications’ side effect, genetic traits, change of smoking habit or sleep-deprivation can also be another source of obesity [2,3,4]. Obesity in turns, is one of the main factors leading to several other health risks such as cardiovascular diseases, hypertension, or cancer [5]. Therefore, it is essential to keep monitoring health parameters in a daily routine to control the energy intake, the type of food, and balance with physical activities to reduce the obesity risks.

Among relevant parameters, glucose and body fat are both important indicators of obesity and able to be acquired daily with numerous developments of non-invasive or minimally invasive monitoring methods [6,7]. The currently, widely used method for home-based glucose monitoring is a glucometer, which draws a small amount of blood from the fingertip using a tiny lancet and quantifies the glucose concentration via a test trip. This method gives a reasonable accuracy but suffers from the risks of infection [8]. As a result, non-invasive monitoring of glucose research has been of interest for many decades with the aim of producing equivalent accuracy while being safe and easy to use. Technically, one can measure the glucose through its concentration in the blood using the light scattering effect, ultrasound or impedance spectroscopy, all of which are directly influenced by the changing glucose concentration, or

through the chemical bond detection or reaction using near-to mid-infrared spectroscopy or Raman spectroscopy [6]. Each method has its own disadvantage and may not yet be comparable to a glucose laboratory test but satisfies enough to be applicable for daily monitoring [6]. Meanwhile, the body fat is mostly analyzed via simple methods such as skinfold thickness measurements using a caliper or ultrasound to quantify the thickness of the subcutaneous adipose tissue, the body mass index and the body circumferences measured at the waist, hip, or upper thigh [7]. These methods however produce certain biases making them unreliable to be used as a standalone parameter but rather being analyzed altogether. Bioimpedance is another method to quantify the body fat, which has gained popularity due to its better correlation to the body fat [7].

With the increasing demand for home-based monitoring devices for self-monitoring one’s health status, non-invasive methods that can be packed in a small size, perform measurement at convenient spots and are easy to use are preferred [9]. As we focus on the body fat and glucose measurement in this study, bioimpedance seems to be a promising method to monitor both parameters. For the glucose measurement, the dominant method was to measure the impedance fluctuations following the blood circulation at the upper arm’s extremity, where a change in the glucose concentration is expected to correlate with the impedance fluctuation’s amplitude [10,11,12]. According to Jingzhen Li et al. [10], the conductivity of the measured impedance was much more sensitive to the varying blood glucose concentration in the range of 1 kHz to 1 MHz than the permittivity. In another study [12], the measured frequency is recommended to be below 40 kHz for stable glucose estimation.

Regarding body fat, the impedance is often measured in the trunk between 2 arms or 2 legs or 1 arm and 1 leg, from which different body compartments are calculated, i.e. the body cell mass, fat mass and fluid compartment [13,14,15]. The body compartments’ estimations are done by assuming the measured trunk impedance as a cylinder model with different layers of adipose tissue (outermost), muscle (middle) and fluid (innermost), from which the amplitude and phase of each layer are calculated [16]. The trunk impedance can be analyzed at one frequency, mostly 50 kHz, or at multiple frequencies ranging from 5 kHz to 1 MHz [16].

There is another extensive study that explained and reviewed the dielectric response caused by the glucose metabolism in the blood [34], and it recommended that the frequency should be much higher, i.e. in the range of MHz to GHz, in order to observe the glucose concentration in the blood in the γ-dispersion. While the glucose bioimpedance measurements gave some preliminarily results with promising accuracy [10,11,12], the body fat is often underestimated by the body compartment bioimpedance method in the obese population due to increasing differences between the assumed cylinder model and the body’s uneven circumferences within the measured area [14,15].

In this research, we aim at a different approach of bioimpedance to estimate both the body fat and the glucose at the same measured spots. Specifically, instead of recording the blood flow related impedance at the upper arm’s extremity, we estimate the glucose indirectly through its relationship with the fat distribution at one part of the body. According to Ahmed H. Kissebah et al. [17], the body fat distribution was found to affect the metabolic aberrations with people having dominantly upper body obesity suffering from increasing instant glucose and insulin levels after oral glucose, more than people having dominantly lower body obesity. The abdominal adiposity was found to consistently relate to the blood glucose [18], while thigh subcutaneous fat is also positively associated with insulin sensitivity and thus, indirectly related to the blood glucose [19]. Therefore, it is possible to estimate glucose through fat content at different body parts. While all the mentioned studies measured fat through X-ray absorptiometry or CT, there is no research up to now measuring the subcutaneous fat locally through bioimpedance and exploring the correlation between the local fat-related impedance and the blood glucose. The local impedance measurement might also be able to compensate for the errors in the cylinder model used by the trunk impedance method, but no research has been done on finding the relationship between the local fat at different parts and the total body fat content.

As a result, this paper will present a bioimpedance measurement method at local body parts and estimate both the body fat and blood glucose. Specifically, we aim to (1) propose a band-shaped bioimpedance system that is battery run, portable, lightweight, easy to implement at different body parts, and convenient, and (2) explore the relationship between the local bioimpedance values and the body fat and blood glucose.

The measurements were done in 4 locations: the upper arm, the belly, the back and the thigh. The impedances were measured at 1 kHz and stored remotely for analysis. The body fat and blood glucose were recorded using a commercial Bioelectrical Impedance Analysis (BIA) and a glucometer respectively. The reasons for picking a frequency of 1 kHz for the impedance measurement are: (1) since the local fat contents are the focus of the study, the conductance of the object would be a more suitable parameter than the capacitance to quantify a fat layer’s thickness and a low frequency would help maximize the conductance measurement, (2) the impedance’s range of 1 kHz-1 Mhz [10,12] is also most sensitive to the glucose changes, and (3) a low frequency would help to make the design more robust, low-power and capable of monitoring in real-time over a long period.

## Materials and methods

### System architecture

**Fig. 1:**
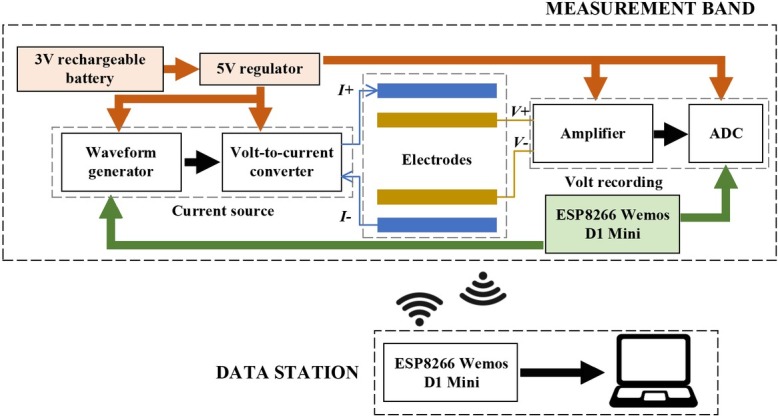
The overall architecture of the system.

Figure 1 presents the overall architecture of the system, which includes the research’s main object, the measurement band, and a data station to store the data for further analysis. Since we aim for a portable band with a reasonable size, the selections of components are kept at minimum specifications including the number of necessary modules, the size of the PCB needed to accommodate each module and the power consumption. The impedance will be measured using the 4-terminal protocol, i.e. the current is injected into 2 electrodes and the voltage is recorded on another 2 electrodes from which the impedance is calculated. The 4-terminal measurement can help avoid the inclusion of the electrode-skin contact impedance in the measurement, which fluctuates among measured positions and people depending on the skin condition and can rise steeply if the contact is poor enough, thus generating significant errors to the impedance measurement. There will be 2 main modules in the measurement band: a current source to drive a stable current to the 2 outer electrodes, and a voltage recording on the 2 inner electrodes. The current source contains a waveform generator to generate a sine wave of 1 kHz and 0.6 V_p-p_ and a voltage-to-current converter to convert the waveform volt into the current of 0.6 mA_p-p_ to be injected into the body. The two voltage lines from the inner electrodes will be amplified using an instrumentation amplifier before being converted to digital at the sampling frequency of 30 kHz for further processing. The controlling and signal processing will be done by a ESP8266 Wemos D1 Mini, which is a tiny board providing just enough pins to control while being fast enough to process the data in between the signal acquisition period, explained shortly. The whole band will be powered by a single supply 5V regulated from a 3.7 V rechargeable battery cell (LIR2032, 70 mAh, 20 mm in diameter, Girafus, Germany).

Meanwhile, the data station is designed minimally to receive the data and store them onto a laptop. The data station also have one command to be sent to the band to repeat the measurement as we aimed to measure several times at each location during the experiment, which will be explained later.

**Fig. 2:**
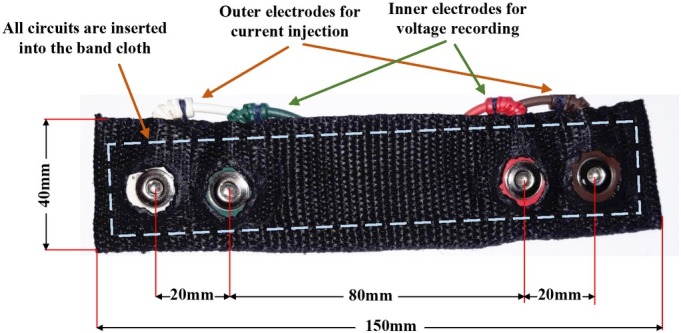
The band prototype of 150mm x 40mm in dimension and the distances between.

The measurement band is designed to fix distances between electrodes to guarantee a consistent impedance measurement at different locations and among different people. Figure 2 exhibits the prototype of the band with the distances of 20 mm between an outer electrode and an inner electrode and of 80 mm between two inner electrodes. The total dimension of the band is 150 mm x 40 mm. All the modules presented in Figure 1 are designed in separated small pieces with soft wires connecting each other, with all inserted into the band cloth. With such breakout small pieces, the band will be able to bend following the skin curve at different locations. In this paper, we used wet electrodes for stable impedance measurements and to study the correlation between the impedances at different sites on the body and the body fat and glucose level.

### The band’s circuit design

**Fig. 3:**
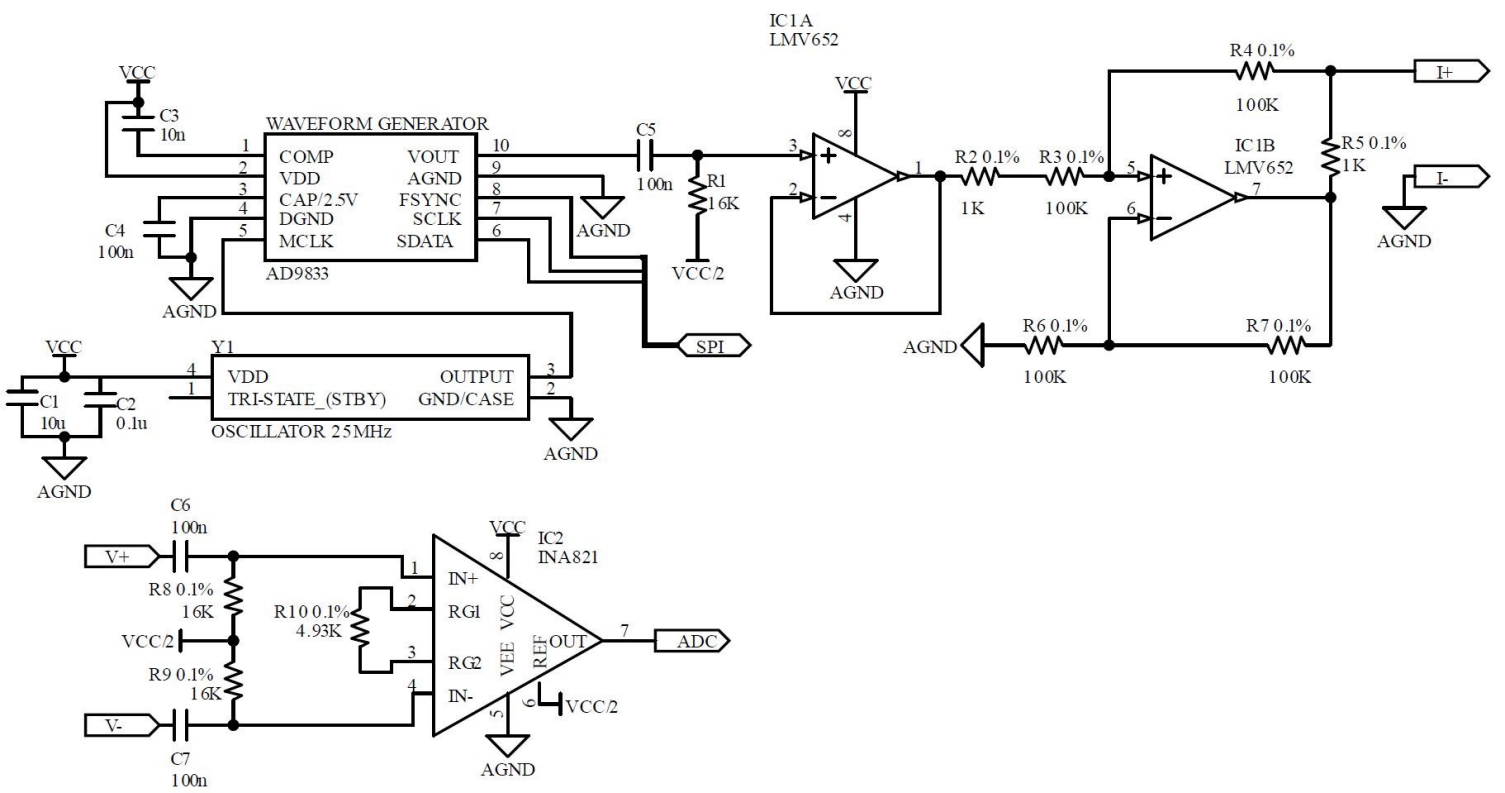
The schematic of the current source and voltage recording on the band


Figure 3 illustrates the circuit design of the current source and voltage recording modules. Specifically, the waveform generator is designed using AD9833 (Analog Devices, USA), a low-power, programmable DAC generator capable of adjusting the frequency up to 12.5 MHz via SPI protocol. The AD9833 consumes little power (quiescent current of 4.5 mA) and produces high SNR (60 dB), low harmonically distorted (THD = −66 dB) sinewave with a fixed amplitude of 0.6 V_p-p_ and adjustable frequency, which was selected to be 1 kHz in this paper. The chip receives the clock signal from an oscillator of 25 MHz. The output waveform is then shifted to the middle point (VCC/2) via a first-order high pass filter (C5 and R1) with the cut-off frequency of 100 Hz before being buffered (IC1A) and converted to the current using the modified Howland current topology (IC1B) [20]. The Howland current topology converts a voltage to a current by adding positive feedback and balancing the resistors on both lines, ideally as:

(1) $$\frac{R_{2}+R_{3}}{R_{4}+R_{5}}=\frac{R_{6}}{R_{7}}$$

Due to the positive feedback, the matching resistances on both lines are vital to avoid voltage surges at the output and maintain a high output impedance for the current source to produce a stable current to variable loads. With high precise resistors of 0.1% as shown in the figure, the current source will in theory be stable to a load up to a few MΩ. In practice, the maximum load for this circuit with 5 V single supply is only around 8 kΩ, where the output will be saturated before it is unstable. Since the wet electrodes would be used here, the electrode-skin contact impedance is only a few hundred ohms with good contact at 1 kHz [21,22], which is entirely within the maximum load capacity (8 kΩ) of the current source. With ideal matching resistors and an ideal case of operational amplifier, the output current can be calculated as:


(2) $$I_{out}=\frac{V_{\text{in }}}{R_{5}}$$

A dual operational amplifier LMV652 (Texas Instruments, USA) is selected for the buffer and the current source. With its high gain-bandwidth (12 MHz), reasonably high input impedance (around 40 MΩ) and low power (quiescent current of 120 μA), the amplifier can be considered to be ideal for the equation (2) to be true, while being suitable for a battery run circuit.

The voltage recording contains an AC bias circuit (C6, C7, R8 and R9) with 100 Hz cut-off frequency and an instrumentation amplifier INA821 (Texas Instruments, USA) to record the differential voltage between the 2 inner electrodes. The resistors and capacitors of the AC bias circuit are precision ones (0.1% resistors and 5% capacitors) to minimize the different phase shifts leading to deteriorated common-mode rejection ratio (CMRR) of the whole measurement module. INA821 is a low-power (650 μA), high gain bandwidth (4.7 MHz), low offset voltage (10 μV), high CMRR (112 dB at the gain of 10), and very low output impedance (1.3 Ω at 10 kHz) IC, which is suitable to record the voltage and drive the ADC IC afterwards. The amplifying gain is set at 10 to accommodate the impedance up to 500 Ω.

The ADC, not shown in the figure, is designed using ADS8320 (Texas Instruments, USA) with low power consumption (1.8 mW at 100 kHz sampling frequency), 16-bit resolution, and low noise (20 μV_rms_).

### Signal processing

**Fig. 4:**
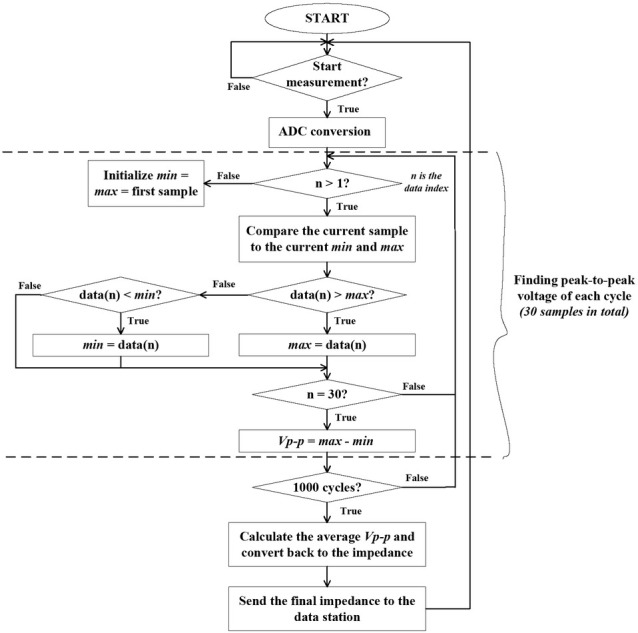
Flowchart of the signal processing

Figure 4 presents the flow of the signal processing. Firstly, the band will wait for a trigger signal from the data station to start a new measurement. If a trigger signal is received, the analog impedance signal will be sampled and converted to digital data with the sampling frequency of 30 kHz. 1000 sine cycles in total will be sampled to deal with the random noise and the powerline noise, leading to 1000 cycles ∗ 30 data points/sample ∗ 16 bits/data point = 60 kBytes data to be stored and sent. Sending wirelessly all 60 kBytes data is not an optimal choice due to a surging power consumption for each transmission, thus, requiring a trimming method to reduce the amount of data necessary to be sent. At the frequency of 1 kHz, the injected current mostly flows in the extracellular fluid leading to the resistance being the only prominent component in the measurement while the capacitance component of the cell membrane is insignificant [23]. Therefore, only the signal’s amplitude is concerned while the phase shift can be ignored.

As a result, the corresponding 1000 peak-to-peak amplitudes are calculated from 1000 cycles and averaged to effectively flatten the noise, especially the powerline noise, as follows:


(3) $${\bar{V}}_{p-p}=\frac{\sum_{i=1}^{1000}V_{p-p}(i)}{1000}(v)$$

where $V_{p-p}(i)\text{ and }{\bar{V}}_{p-p}$are the individual peak-to-peak amplitude (i = 1 to 1000) and the final averaged amplitude, respectively.

The calculation of each peak-to-peak amplitude is performed and updated in between two consecutive ADC conversions with a simple method shown in Figure 4. Specifically, two variables, *min* and *max*, representing the minimum and maximum values in one cycle, are initialized as the first sampled point at the beginning of each cycle. When a new point arrives, *min* and *max* are updated by comparing their previous value with the current point. In this way, the absolute minimum and maximum points can be found without confusing different peaks and valleys in a distorted cycle due to noise. This method is also fast to be adopted in between two sampling times. When a full cycle is sampled, i.e. the 30^th^ point arrives, the amplitude $V_{p-p}$will be the difference between *min* and *max*. This process is repeated until sampling all 1000 cycles, the averaged voltage amplitude is then calculated and converted to impedances as:


(4) $${\bar{R}}_{\text{local }}=\frac{{\bar{V}}_{p-p}}{2*\alpha }$$

Where ${\bar{R}}_{\text{local }}$is the final impedance measured at one location and αis a conversion factor. The factor αcan be estimated given the known current amplitude and amplifying gains from the circuit. However, any errors from the components and the PCB can be accumulated in the αcalculation. Therefore, we instead calibrated αwith 2 resistors (0.1% precision) 100 Ω and 470 Ω and obtained α=2.705 ∗ 10^−3^. Only the final impedance ${\bar{R}}_{\text{local }}$is sent to the data station instead of 60 kBytes raw data at the beginning, thus optimizing the power consumption and allowing the band to run several hours with only a 70 mAh battery.

## Experiment design

### Testing with resistors

A small test was designed to test the accuracy of the final averaged impedance ${\bar{R}}_{\text{local }}$calculated from the system when compared with standard resistors. 9 resistors of 0.1% precision were selected for the test including 10 Ω, 22 Ω, 39 Ω, 47 Ω, 68 Ω, 100 Ω, 220 Ω, 390 Ω, and 470 Ω. Any significant errors between the measured ${\bar{R}}_{\text{local }}$and the tested resistors would indicate the system’s inefficiency in treating noise that makes the measurements fluctuate.

### Measurements on volunteers

**Fig. 5:**
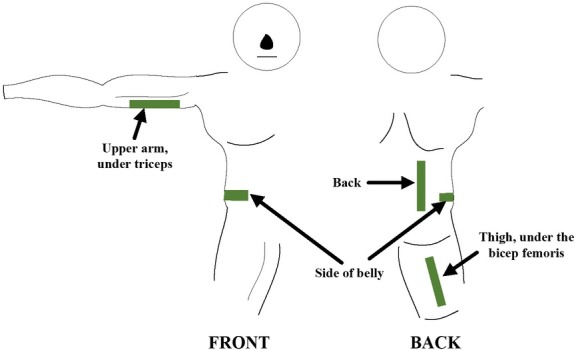
Four locations were picked for the local impedance measurement: on the upper arm under the triceps, on the side of the belly, on the side of the back and on the thigh under the bicep femoris

A total of 21 volunteers, all male, agreed to join the experiment and were recruited at Hanoi University of Science and Technology. 9 of them were diagnosed with diabetes. Table 1 presents the information of the volunteers, where the body fat (%) and the blood glucose level were obtained using a bioimpedance weight scale BF7000 (Beurer, Germany) and a glucometer Accu-Chek (Roche, Switzerland), respectively. The volunteers were asked to fast at least 8 hours before the next morning when the glucose level was measured. The band was placed at 4 different locations shown in Figure 5: the upper arm under the triceps, the side of the belly, the back on one side and the upper thigh under the bicep femoris. Those locations were picked because the subcutaneous fat often accumulates there. The corresponding 4 local impedances would be measured at those locations 10 times and their correlations to the body fat and the fasting glucose level would be analyzed to find out how feasible the impedance measurement method is in estimating the two parameters and which location gives the best results in estimating either of them. The Spear correlation coefficient between the final averaged impedance and the body fat and glucose, and the errors (mean ± standard deviation) between the values predicted by the linear lines from the impedances and the measured ones were calculated for the evaluation.

**Table 1:** The information of volunteers in the experiment including age, BME, body fat (%) and fasting glucose

	Age	BMI	Body fat (%)	Fasting glucose (mmol/l)
***mean ±***	31.9 ±	22.1 ±	20.7 ± 6.9	7.4 ±

***std***	6.6	3.5		1.8

***min***	18	17.3	6.2	4.9

***max***	44	27.6	33.1	10.9

### Informed consent

All volunteers in this study had given their informed consent to the experiment conduction.

### Ethical approval

The research was tested on human who volunteered to join the experiment at Hanoi University of Science and Technology, Vietnam. There was no institutional review board or equivalent committee at experimenting site, but the research has been carefully performed to be complied with all relevant national regulations, institutional policies and in accordance with the tenets of the Helsinki Declaration.

## Results

### Testing with resistors

**Fig. 6:**
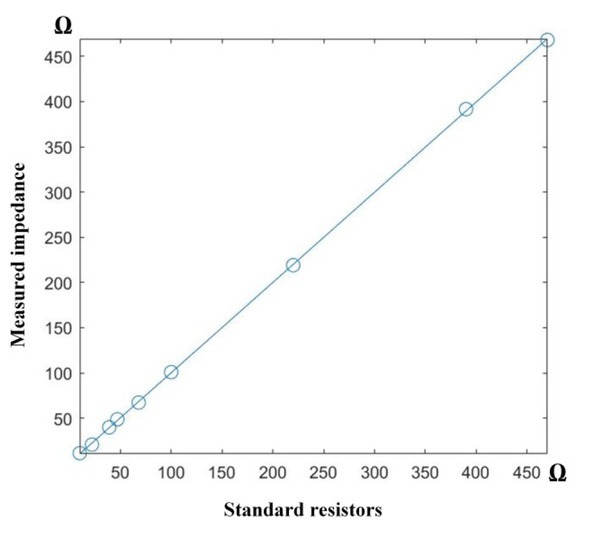
Results between the impedance measured by the band and the standard resistors

Figure 6 exhibits the results of measured impedances versus the standard resistors, showing the linear line being mostly diagonal with very low errors (1.2 Ω ± 0.4 Ω). In other words, averaging 1000 cycles has helped flatten all noises efficiently and thus, the system’s accuracy was approved for the next impedance measurement on volunteers.

### Measurements on volunteers

**Fig. 7:**
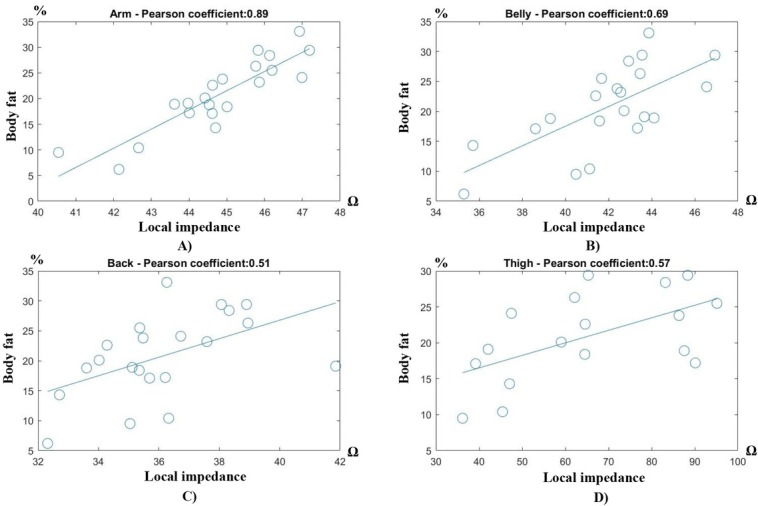
Local impedance versus body fat at 4 locations: A) upper arm, B) belly, C) back, and D) thigh.

**Fig. 8:**
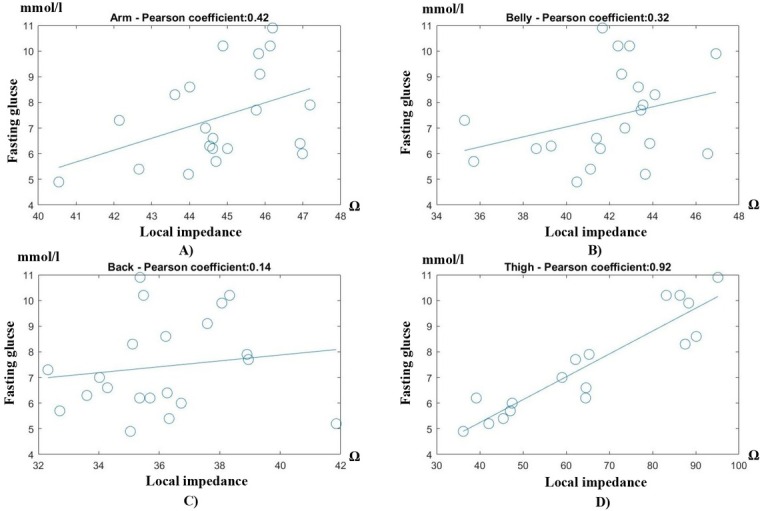
Local impedance versus fasting glucose level at 4 locations: A) upper arm, B) belly, C) back, and D) thigh.

Out of 21 volunteers, we have totally 21 impedance measurements on the upper arm, the belly and the back, and 16 measurements on the thigh due to 5 volunteers refusing to take off the pant for the thigh measurement. The correlation coefficients and their corresponding confidence interval - CI (95%) between the local impedances and the body fat and fasting glucose are presented in table 2, while Figures 7 and 8 exhibit the detailed correlation results to the body fat and the glucose level, respectively. As can be seen, there was a significant agreement between the impedance measured at the arm under triceps and the body fat with the correlation coefficient of 0.89, and between the impedance at the thigh under the bicep femoris and the fasting glucose level with the coefficient of 0.92. The CIs in the arm impedance-body fat and the thigh impedance-glucose correlations were also tight (95% CI of 0.73-0.95 and 0.81-0.97 for fat and glucose correlations, respectively), showing that the impedances at the arm and thigh are highly reliable indicators of the body fat and fasting glucose. Reversely, the arm impedance is not as good an indicator of the glucose (coefficient of 0.42) as the thigh impedance to the body fat (coefficient of 0.57). Similarly, both the belly and the back impedances have a medium correlation to the body fat (coefficients of 0.69 and 0.51 respectively) while being very poor in the glucose prediction (coefficients of 0.32 and 0.14 respectively).

**Table 2:** Correlation coefficients and the corresponding confidence intervals (95%) between the local impedances measured at 4 locations: the upper arm, the belly, the back and the thigh, and the two parameters: the body fat and fasting glucose of all 21 volunteers. The best results are highlighted in boldness and red. **Abbreviation:** *Cor. Coef*: correlation coefficient. *CI*: confidence interval (95%)

	Arm		Belly		Back		Thigh	
	
	*Cor*. *Coef*.*	CI**	*Cor*. *Coef*.*	CI**	*Cor*. *Coef*.*	CI**	*Cor*. *Coef*.*	CI**
**Body fat**	**0.89**	**0.73-0.95**	0.69	0.36-0.86	0.51	0.09-0.77	0.57	0.13-0.82

**Fasting Glucose**	0.42	-0.02-0.72	0.32	-0.14-0.65	0.14	-0.31-0.54	**0.92**	**0.81-0.97**

In general, the local impedances seemed to be correlated better to the body fat than the fasting glucose. The average coefficient of the impedance-body fat correlation at all locations was 0.66 while that of the impedance-glucose level was only 0.42, as shown in figures 7 and 8. It shows that the body fat component was generally better correlated to the local impedance than the glucose. However, in terms of individual correlation, the thigh impedance was able to predict the glucose more reliably (95% CI < 0.11) than the arm impedance to the body fat (95% CI < 0.16).

Regarding the locations, the upper front body (arm and belly) was more suitable for predicting the body fat (correlation coefficient of 0.79 in average as in figure 7), while the lower body (thigh) was better for the fasting glucose prediction (correlation coefficient of 0.9 as in figure 8). Meanwhile, measurements on the back were not favored for both the body fat and the glucose predictions (correlation coefficients of 0.51 (figure 7) and 0.14 (figure 8), respectively).

## Discussion

### Main findings

In this study, a portable band-shaped bioimpedance system was designed and tested on 21 volunteers. From the results, two main findings were found including:

- The bioimpedance band has successfully achieved stable measurements when being applied in different body parts from the upper to the lower body. With low current injection (only 0.3 mA in amplitude at 1 kHz), there were no complaints of discomfort and inconvenience from the volunteers while using the band.
- The local impedances were found to be well correlated with the body fat (measured on the upper arm under the triceps) and the fasting glucose level (measured on the thigh under the bicep femoris), with both correlation coefficients of around 0.9 with 95% confidence interval of 0.73-0.95 for the body fat and 0.81-0.97 for the glucose. This proves that the relationship between the local impedance and the two health parameters were significant enough to allow predictions with reasonable accuracy.

### Local impedance correlation – body fat versus fasting glucose

From figures 7 and 8, it can be observed that the impedance-body fat correlation was generally higher than the impedance-glucose correlation at all measured locations. This is understandable since the glucose is measured indirectly through the subcutaneous fat’s impedance which can be affected by many other factors aside from the glucose metabolism. Individually, the body fat was best predicted when the local impedance was measured on the upper arm under the triceps, while the thigh under the bicep femoris would be the best location for the fasting glucose prediction. More interestingly, the impedance-glucose correlation measured on the thigh was better and more reliable (0.92 coefficient, 95% CI: 0.73-0.95) than that of the impedance-body fat measured on the thigh (0.89 coefficient, 95% CI: 0.81-0.97). This result is unexpected but promising to show the system’s capability in predicting the fast glucose level with low errors. However, the upper arm local impedance – body fat prediction was not far behind, which we believe still is a reasonable error since the glucose is the more important parameter and a fluctuation in the fasting glucose affects the health more detrimentally than a fluctuation in the body fat.

### Body locations on the body fat and glucose predictions

From table 2,figures 7 and 8, it can be seen that the upper front body (arm and belly) was the better location to predict the body fat, while the lower body (thigh) was the candidate for the glucose prediction. The results show a positive correlation between the arm impedances and the whole body fat (%), meaning a higher arm impedance, i.e. thicker fat layer, should lead to a higher body fat. This is in line with some other studies using X-ray anthropometry [24] and cylindrical modelling impedance [25] where a similar positive correlation between the upper body’s subcutaneous fat and the whole body fat was confirmed. However, the correlation between the fat contents on the thigh and the blood glucose was more complicated. According to our results, there was a strong positive thigh impedance – fasting glucose correlation, but many studies revealed a reversed trend where the thigh’s subcutaneous fat had a negative correlation with the blood glucose, meaning an accumulation of the subcutaneous fat on the thigh is actually protective against insulin resistance [19,26,27,28,29,30]. However, another of thigh’s fat content called the intermuscular adipose tissue (IMAT), the fat tissue underneath deep fascia and between muscle groups, is the tissue that positively correlates to the blood glucose [26,30,31,32]. Some studies [30,32] also concluded that a combination of low subcutaneous fat and high IMAT or visceral fat measured on the thigh is a much better indicator of an increasing insulin resistance. Unfortunately, in this study we could not access the X-ray facility to perform dual X-ray absorptiometry or CT to quantify different fat contents on volunteers. From our subjective observation from the outside, our volunteers (all Asians) including the ones diagnosed with prediabetes do not have excessive thigh’s subcutaneous fat, which actually agrees with a study [33] showing that Asians have less subcutaneous fat on the thigh. Since the measurement band is large enough (40 mm width x 150 mm length) to cover at least two-thirds of the thigh’s length, the current is likely able to penetrate deeper beyond the deep fascia and thus, the thigh impedances might cover a considerable portion of intermuscular fat apart from the subcutaneous fat layer, which possibly explains the positive correlation between the thigh impedances measured by the system and the blood glucose. This requires an extensive trial on a much larger samples to confirm, which will be conducted in a future study.

Among the two parameters, except for the arm, the body fat was predicted with equally average accuracy (coefficient of 0.5-0.7) when measuring the impedances on both upper and lower body parts. It shows that the body fat is an easier parameter to be predicted with the local impedance, but the accuracy is not enough showing that there might be other factors contributing to the measured impedances at the thigh, back and belly that reduce the body fat prediction in overall. Within this experiment, the upper arm under the triceps remains the best spot to predict the body fat.

### Study limitations

The first limitation of this study is the number of measured subjects. Twenty-one volunteers with the same number of impedance measurements on the upper arm, the belly and the back and even less on the thigh are not statistically enough and likely susceptible to the random variations that does not allow to draw a concrete conclusion on the performance of one method. Nonetheless, we managed to collect a wide range of fasting glucose levels and body fat from normal to prediabetes and diabetes. Therefore, the obtained results are still qualified to evaluate the feasibility and, to some extent, the capability of the proposed system in measuring the two parameters. Future clinical trials will be conducted to confirm the reproducibility of these results.

Another limitation is the methods to obtain the ground truth of both the fasting glucose and the body fat. Due to limited access to laboratory facilities, we could only use a glucometer whose accuracy cannot match that of a blood test and thus, is not the best means for the comparison. We were also not able to access any X-ray facilities to quantify the body fat using X-ray anthropometry or CT. The weight scale used for measuring the body fat in the study is actually another bioimpedance method and its accuracy is not of gold standard. Nonetheless, with promising correlation between the local impedances and the glucometer’s the bioimpedance scale’s results can be used as evidence for the research group to apply for clinical trials in the future where blood tests will be available.

## Conclusion

In this paper, a portable battery-run bioimpedance band was proposed for the home-based non-invasive measurement of body fat and fasting glucose. The details of the hardware design and the signal processing were presented. The proposed system was tested on the volunteers and we obtained promising results with the arm under the triceps being the best location to predict the body fat, and the thigh under the bicep femoris for the fasting glucose prediction. These preliminary results indicate a feasibility and capacity of the proposed system as a home-based, portable and convenient system targeted for monitoring the body fat and glucose. The performance in predicting body fat and glucose will be verified and replicated in a future, larger study.
